# Beyond Tanimoto: a learned bioactivity similarity index enhances ligand discovery

**DOI:** 10.3389/fbinf.2025.1695353

**Published:** 2025-11-28

**Authors:** Gustavo Schottlender, Juan Manuel Prieto, Marcelo A. Marti, Dario Fernández Do Porto

**Affiliations:** 1 Instituto de Cálculo, Facultad de Ciencias Exactas y Naturales, Universidad de Buenos Aires, CONICET, Universidad de Buenos Aires, Buenos Aires, Argentina; 2 LUCAI BIO, Dover, DE, United States; 3 Departamento de Química Biológica, Facultad de Ciencias Exactas y Naturales, Universidad de Buenos Aires (FCEyN-UBA), Buenos Aires, Argentina; 4 Instituto de Química Biológica de la Facultad de Ciencias Exactas y Naturales (IQUIBICEN) CONICET, Pabellón 2 de Ciudad Universitaria, Buenos Aires, Argentina

**Keywords:** bioactivity similarity index, BSI, machine learning, molecular embedding baselines, ChemBERTa, clamp, virtual screening

## Abstract

Structural similarity metrics such as the Tanimoto coefficient (TC) miss many functionally related compounds—indeed, 60% of similarly bioactive ligand pairs in the ChEMBL database show TC < 0.30, revealing a major blind spot that constrains ligand-based discovery. Our motivation is to overcome this blind spot and enable the recovery of structurally different yet functionally equivalent chemotypes that structure-based similarity fails to detect. Here, we introduce the bioactivity similarity index (BSI), a machine learning model that estimates the probability that two molecules bind the same or related protein receptors. Trained under leave-one-protein-out (LOPO) across Pfam-defined protein groups on dissimilar pairs, BSI not only outperforms TC but also surpasses modern molecular embedding baselines (ChemBERTa and contrastive language-molecule pre-training (CLAMP), using cosine similarity) across protein families. We further develop a cross-family model (BSI-Large) that, while slightly below group-specific models, generalizes better and can be fine-tuned with less data, consistently improving over models trained from scratch. In retrospective validation on new ChEMBL v35 data, BSI achieves strong early-retrieval performance (top 2% enrichment factor, EF_2%_), with group-specific models delivering the best enrichment, and BSI-Large remaining competitive. In a realistic virtual screening-like scenario against the target gene ADRA2B, the mean rank of the next active, given a known active, improves from 45.2 (TC) to 3.9 (BSI), with 54.9 for ChemBERTa and 28.6 for CLAMP. Altogether, BSI complements, rather than replaces, structure-based similarity and embedding-based comparisons, extending hit finding to remote chemotypes that are structurally dissimilar yet functionally equivalent. The code is available at https://github.com/gschottlender/bioactivity-similarity-index.

## Introduction

Developing new therapeutic drug-like compounds remains a central challenge in modern biomedicine, particularly in the face of increasing antimicrobial resistance and the high attrition rates in drug discovery pipelines. A critical step in this process is identifying chemical compounds with relevant and desired biological activities that can lead to novel therapeutic drugs down the clinical research pipeline. These lead compounds can be identified from purely experimental or virtual screening (VS) campaigns combined with experimental validation. A powerful strategy for selecting candidates is based on the assumption that structurally similar molecules will bind to the same protein and exhibit similar biological effects. This principle underlies, for example, the well-known use of substrate analogs as enzyme inhibitors. The chemical similarity strategy is further bolstered by the decades-long observation that structural similarity of compounds usually results in biological function similarity (Nikolova and Jaworska, 2003; Maggiora et al., 2014). Central to the strategy mentioned above is the following question: How can we effectively compare small molecules?

Over the last few decades, the dominant strategy has been to assess molecular resemblance using structural fingerprints, computing the so-called chemical similarity indices such as the Tanimoto coefficient (TC) (Bajusz et al., 2015). Although this type of approach has proven highly effective for predicting bioactivities of interest (Schuffenhauer et al., 2006; Chen et al., 2015), it inherently focuses on chemical features rather than directly capturing the underlying molecular mechanism resulting in its biological effect (Safizadeh et al., 2021; Fernández-Torras et al., 2022), limiting its ability to detect compounds with similar bioactivities but divergent structures (Martin et al., 2002). Moreover, a substantial fraction of functionally related compounds remains invisible to structure-based comparisons. In particular, many similarly bioactive ligand pairs in large public resources (e.g., ChEMBL) fall below conventional similarity cutoffs (such as TC < 0.30), creating a blind spot that constrains ligand-based discovery and the exploration of structurally remote chemotypes.

This issue is particularly pressing in the antimicrobial discovery and development field, where the need for innovation has driven the integration of genomics, structural biology, and computational methods to improve target prioritization and lead discovery (Sosa et al., 2018; Arcon et al., 2021; Serral et al., 2021; Serral et al., 2022; Marti et al., 2024). In this context, new strategies that go beyond structural similarity strategies that result in the generation of new drugs harboring the same chemical scaffold (for example, beta-lactams) but instead focus on the prediction of compound bioactivity represent a promising research endeavor.

Most drugs exert their effect by binding tightly to a given protein target and modulating its activity, and as already mentioned, similar compounds usually bind to the same protein. Moreover, similar proteins also usually bind the same compound, and the combination of both observations results in similar compounds binding to similar proteins. Leveraging on this “guilt-by-association” principle and TC, in our previous study (Radusky et al., 2017), we demonstrated that significant enrichment of true binders can be achieved in the context of virtual screening. Moreover, this approach allows for the identification of potential molecular targets for compounds found to be active in phenotypic screens (Schottlender et al., 2022), as implemented in the platform available at https://github.com/gschottlender/ReverseLigQ.

Both these applications are constrained by the method used to compute chemical similarity. However, it is a well-established fact that some ligands that bind with similar strength to a given target can differ substantially in their chemical structures (Ghosh et al., 2012; Wang et al., 2018). In this scenario, we hypothesized that it could be possible to predict whether two molecules bind similarly to the same (or a related) target, without relying on conventional chemical similarity metrics. Such an approach could substantially broaden the scope of the aforementioned strategy in a VS context. To this end, we leveraged the growing availability of public protein–ligand-binding data and recent advances in deep learning.

Recently, deep learning (DL) strategies have achieved remarkable progress in the life sciences, offering powerful tools for modeling complex patterns in biological and chemical data. These algorithms have been successfully applied to predict molecular properties (Feinberg et al., 2018; Walters and Barzilay, 2021; Pang et al., 2023), uncover nonlinear relationships between structure and biological activity (Jeon et al., 2021; Prajapati et al., 2025), and model protein structures and their interactions with small molecules (Abramson et al., 2024; Zhang et al., 2024). In the drug discovery field, deep neural networks offer the potential to overcome some of the limitations of structure-based similarity comparisons by more accurately capturing the subtle correlations between chemistry and bioactivity. Therefore, we decided to explore whether a DL architecture could capture the underlying similarity of the binding capacity of chemically diverse compounds.

In this context, our motivation is to recover structurally dissimilar yet functionally equivalent compounds, thereby expanding the discovery space and reducing screening burden. We therefore present the bioactivity similarity index (BSI), a deep learning-based method that compares pairs of molecules and estimates a bioactivity-centered similarity—that is, the probability that they bind to the same or related protein targets. BSI recovers and enriches functional analogs at low levels of structural similarity (e.g., TC < 0.30–0.40) across protein families and supports transfer learning for underrepresented families through the fine-tuning of models trained on multiple families. Its current scope has limitations: performance is protein-group dependent with limited generalization to unseen families; fingerprint tokenization, while cost-efficient, may be suboptimal relative to more complex molecular representations and should be systematically evaluated in future work; and training relies on a finite labeled universe (e.g., ChEMBL), implying the need for fine-tuning or domain adaptation in different real-world scenarios. Accordingly, we position BSI as a complement—rather than a replacement—to conventional structure-based metrics and embedding-based similarities.

Our results show that BSI outperforms similarity comparisons between two modern state-of-the-art molecular representations (ChemBERTa and contrastive language-molecule pre-training (CLAMP)) in identifying compounds that share protein targets when they are structurally dissimilar.

Finally, we propose that the described method can be applied to clinically important protein groups, regardless of the specific target evaluated, and serve as a starting point for the development of more sophisticated tools for comparing compounds based on their bioactivities.

## Materials and methods

### Retrieval of compounds from ChEMBL

All compounds with reported bioactivity, either characterized by a pChEMBL value (a standardized measure of bioactivity across assay types) (Bento et al., 2014) or otherwise annotated with a bioactivity comment, were retrieved through SQL queries from the ChEMBL database (version 33) (Zdrazil et al., 2024). For each compound, we retrieved its SMILES representations (Wigh et al., 2022), the corresponding protein targets (represented as UniProt IDs) (UniProt Consortium, 2025), and their associated Pfam families (Paysan-Lafosse et al., 2025).

Active or binder compounds were defined as those with a pChEMBL value above 6.5, roughly equivalent to a Ki of 300 nM, according to previously established criteria (Lenselink et al., 2017; Ye et al., 2022). Because experimentally confirmed non-binders are scarce, we defined experimentally verified inactives as compounds with pChEMBL <4.5 (≈ Ki ≥ 30 µM), in line with previous work (Burggraaff et al., 2020). A similar criterion was also adopted by the Directory of Useful Decoys, Enhanced (DUD-E) (Mysinger et al., 2012), which defines them as compounds with no measurable affinity up to 30 μM (corresponding to a pChEMBL value, that is, the negative logarithm of a Ki of 4.52). Additionally, compounds explicitly marked as inactive in ChEMBL bioactivity comments were also included in this group. Because ChEMBL is highly imbalanced toward active compounds, additional inactive compounds (decoys) were built for each target using the DUD-E methodology. Specifically, for every compound that exhibited activity against any ChEMBL target, we generated a set of corresponding decoys. Each decoy is required to have a molecular weight within ±25 Da of the active ligand, a logP within ±1 unit, the number of rotatable bonds within ±2, hydrogen bond acceptors and donors within ±1, and an identical net charge. A chemical similarity threshold (TC < 0.3) was therefore applied in concert with the preceding physicochemical constraints—precisely because compounds with similar bioactivities can also fall below this cutoff, a point that is critical to our study—in order to yield decoys whose bioactivity profiles are expected to diverge from those of the corresponding actives. This literature-supported strategy is clearly preferable to augmenting the dataset with random molecules (Mysinger et al., 2012; Scantlebury et al., 2020).

We finally ensured that the similarity distribution between active compounds and decoys resembled that observed between active and experimentally verified inactive compounds (See Supplementary Figure S1). A two-sample Kolmogorov–Smirnov test confirmed that the distributions of TC values for N pairs (composed of one active ligand and one inactive counterpart) built using the decoys and the distribution of coefficient values of those N pairs built using active and inactive compounds that were both derived from ChEMBL were effectively identical below TC = 0.40 (D = 0.019, *p* < 1 × 10^−300^; n = 5.1 × 10^6^ and 8.6 × 10^6^ pairs, respectively). A Jensen–Shannon divergence of 0.02 further corroborated the negligible disparity between the two curves. Note that a minority of active–decoy pairs exhibits TC > 0.30 because decoys were selected independently for each active ligand of the same protein, so a decoy chosen for one active can display marginally higher, yet still low, similarity to another active ligand. These results show that, within the relevant similarity range, the decoy-based negatives faithfully replicate the statistical properties of experimentally verified inactives.

### General dataset assembly for model training

The models were designed to classify compound pairs into two categories based on their bioactivity: pairs with similar bioactivities (S) and pairs with non-similar bioactivities (N). Therefore, S pairs consist of two molecules that are both active against the same protein target, while N pairs comprise one compound that is active and one compound that is inactive against the same protein.

To mitigate dataset bias due to proteins with a disproportionately high number of active compounds, three clustering methods were sequentially applied. First, Bemis–Murcko scaffold clustering (Bemis and Murcko, 1996) was performed to group compounds by core structure, selecting one representative per cluster. Second, we applied Butina clustering with a TC threshold of 0.4 (Butina, 1999). Finally, if more than 100 compounds still remained for a given protein, K-means clustering (MacCuish and MacCuish, 2014) was used to reduce the number of actives to a maximum of 100 per protein, ensuring a balanced and diverse set of actives for each target. In contrast, inactive compounds for each target were selected according to the DUD-E criteria, as previously described. Due to the limited number of experimentally validated negatives, a data augmentation strategy was applied using decoys, which were individually selected for each active compound targeting the same protein, using the previously explained methodology.

After selecting active and inactive compounds for each protein, S pairs were generated by pairing all active compounds with each other (all-vs-all), while N pairs were formed by pairing each active compound with all inactive compounds. Only compound pairs (S and N) with a Tanimoto coefficient below 0.4 were retained to develop the algorithm on structurally dissimilar pairs, emphasizing bioactivity-centered signal over chemical structural likeness.

Finally, compound pairs were encoded by directly summing their Morgan fingerprints (256 bits, radius 2) (Morgan, 1965; Rogers and Hahn, 2010), as implemented in RDKit (https://www.rdkit.org). We built two types of datasets, one slightly imbalanced (25:75 ratio of S to N pairs) and another heavily imbalanced (4:96 ratio of S to N), by tenfold decoy augmentation. Protein groups with their corresponding targets and corresponding final S pairs are shown in Supplementary Table S2.

### Model training and evaluation metrics

All classification models were implemented as feedforward neural networks using PyTorch (version 2.5.1). The input layer received the combined fingerprint vector, and thus, the first layer has 256 neurons. The final layer is a one-neuron classifier using a sigmoid activation function that predicts the probability of the input (i.e., the compound pair) as belonging to the S or N category (Paszke et al., 2019). All hidden layers used the ReLU activation function. Unless otherwise specified, training was performed using the Adam optimizer with a default learning rate of 0.001, and binary cross-entropy was used as the loss function.

For each training scenario, hyperparameter tuning was carried out to identify the optimal architecture, including the number of hidden layers, dropout probability, and the number of training epochs (Gawehn et al., 2016; Rasamoelina et al., 2020).

### Reference methods for comparison

To place our method in the context of recent advances in molecular representation learning, we compared its performance with two state-of-the-art embedding models using cosine similarity.

We first employed ChemBERTa (Chithrananda et al., 2020), a Transformer-based architecture pretrained on molecular representations encoded from SMILES strings. The ChemBERTa models were implemented using the Hugging Face Transformers framework (Wolf et al., 2019), and molecular embeddings were generated with mean pooling. Three different pretrained versions were evaluated—DeepChem/ChemBERTa-100M-MLM, DeepChem/ChemBERTa-77M-MLM, and seyonec/ChemBERTa-zinc-base-v1. Among them, DeepChem/ChemBERTa-77M-MLM exhibited the highest mean AUC (0.61) across the evaluated major protein groups (MPGs) and was therefore selected for subsequent analyses.

As a second reference method, we evaluated CLAMP (Seidl et al., 2023), a multimodal model trained with contrastive learning to align molecular representations with free-text bioassay descriptions. This makes it a natural baseline to probe bioactivity-aware ligand encodings in our approach. Recent large-scale benchmarks reported a solid performance of pretrained CLAMP embeddings across diverse datasets, outperforming all other recent deep learning-based representations (Praski et al., 2025). We computed CLAMP molecular embeddings using the official implementation (by running the script encode_compound.py) provided in the GitHub repository.

### Predicting bioactivity compound similarity across major protein groups

Given the natural imbalance in the amount of ligand-target information for different protein families, we built different models for different protein groups. We first grouped targets according to protein families as defined in Pfam (Paysan-Lafosse et al., 2025). Families with the largest number of proteins (PF00001, PF00069, and PF07714) were further subdivided into smaller groups based on sequence identity (the corresponding targets belonging to each subgroup are detailed in Supplementary Table S2). These resulting clusters are referred to as MPGs.

We first built BSI models independently for each MPG, using a leave-one-protein-out (LOPO) approach (Høie et al., 2022). Thus, the model was trained on data from all proteins within the group except one, which was used for testing. This process was iterated over all proteins in each group. Evaluation was performed using the ROC and precision-recall (PR) AUCs, as described below.

To identify a reasonable parameter configuration that performed consistently across different biological contexts, three distinct test sets were defined, each corresponding to a specific protein group: PF00069 subgroup A, PF00026, and PF00089.

For each of these test sets, suitable parameter configurations for the MPG models were evaluated using the LOPO approach, by combining three hidden layer architectures ([256], [256, 128], and [256, 128, 64]), three dropout probabilities (0.1, 0.25, and 0.5), and three training lengths (5 epochs, 10 epochs, and 15 epochs), yielding a total of 27 configurations. Additional epochs were not considered as the loss function displayed progressively slower improvement beyond 10 epochs, while further training would substantially increase computational cost and the risk of overfitting. Given that multiple models had to be evaluated under different conditions, this trade-off was considered acceptable.

The configuration that achieved the most consistent performance across all three test sets was a relatively simple one: a single hidden layer with 256 neurons, a dropout rate of 0.5, and 10 training epochs. This combination was ranked 3rd by ROC-AUC on the PF00069 subgroup A dataset, 5th on PF00026, and 7th on PF00089.

In addition, because Morgan fingerprints were initially selected as the reference molecular representation, comparative analyses with the Molecular ACCess System (MACCS) (Joseph et al., 2002) and RDKit fingerprints were conducted (using the predetermined parameter configuration). These alternative representations showed comparable predictive performance, with Morgan fingerprints achieving slightly higher mean ROC-AUC values across the three test sets (0.80 per protein group), compared to 0.78 for MACCS and 0.71 for RDKit fingerprints.

The metrics obtained with the BSI models were compared to those from the reference methods, ChemBERTa and CLAMP (using cosine similarity). Statistical significance was assessed using Student’s t-test to compare the performance of the BSI models against the reference methods.

We also built a general MPG model, referred to as BSI-Large, trained by merging the data of all MPG into a single training set. In this case, the best model hyperparameters were a hidden layer configuration of [512, 256, 128, 64], 10 training epochs, a learning rate of 0.0001, and a dropout of 0.3. BSI-Large performance was evaluated with ROC-AUC using the LOPO approach on four different protein groups: PF00209, PF00413, PF00520, and PF00850.

The numbers of active compounds, experimentally verified inactives, and decoys used in each dataset for model training are detailed in Supplementary Table S3.

### Model generalization assessments

To test the ability of the models to make predictions on protein groups whose data were not included in the training sets, we constructed additional datasets from families containing fewer than 10 protein targets and with reported bioactive compounds in ChEMBL. We refer to these as underrepresented protein groups (UPGs). Each group dataset was generated as previously described, comprising S pairs (containing two active compounds) and N pairs (each consisting of one active compound and one decoy) with a 25:75 ratio for S and N pairs. Performance on the different UPG datasets for models trained on each MPG, as well as for the BSI-Large model, was evaluated using the ROC-AUC.

### Transfer learning assessments on protein groups with limited data

For the transfer learning analysis, we selected the 15 protein groups with the fewest bioactive compounds and at least two targets reported in ChEMBL (a subset from the UPG, referred to as less represented protein groups, LRPGs). For each group, random samples of 99 active compounds were taken to standardize the dataset size across families. Training datasets were then assembled by progressively increasing the number of bioactive compounds in increments of 10. S pairs were constructed using only these bioactive compounds, while N pairs consisted of all active compounds paired with all of the corresponding decoys. For N pairs, the number of bioactive compounds and decoys used was kept equal. Evaluation was performed on the remaining data, ensuring that no compounds were shared between the training and evaluation sets.

The modeling approach consisted first of using the BSI-Large model and performing a fine-tuning with the data from each LRPG and, second, training a control model from scratch with the same architecture. Training was performed using five epochs and a learning rate of 0.0001, without layer freezing for the BSI-Large. Evaluation was carried out using the ROC-AUC to distinguish between S pairs and N pairs within each LRPG, for each number of active compounds used (and a similar number of decoys).

### Validation on new experimentally verified data from ChEMBL v35

Retrospective validation with ChEMBL 35 data was performed as an additional evaluation on previously unseen bioactivity records. Because all our models had been trained on ChEMBL version 33, we first compared this release with ChEMBL version 35 and retained only those records unique to the newer version. The resulting validation set comprised 88 targets distributed across 19 MPGs and 21 targets belonging to 16 UPGs. For every target in this set, we generated compound pairs exactly as previously described, with a deliberately stronger class imbalance to emulate realistic virtual screening conditions. All pairs were required to exhibit a Tanimoto coefficient of less than 0.3.

The evaluation of MPG data employed the full suite of pretrained models—namely, the group-specific BSI models and the global BSI-Large. For UPG data, we created a modified version of BSI-Large that was fine-tuned for five epochs on ChEMBL 33 data from the same 16 UPGs (learning rate of 0.0001, no layer freezing). Performance was quantified with the top 2% enrichment factor (EF_2%_), a metric that directly reflects hit-retrieval efficiency in virtual screening (Ganser et al., 2018). EF_2%_ values obtained with predictions by the models were compared with results based on our reference methods, ChemBERTa and CLAMP, and statistical significance was assessed using Student’s t-test for each evaluated protein group.

As a final case study, we performed a virtual screening (VS)-like validation against α2B adrenergic receptor (ADRA2B; Pfam PF00001 subgroup D), which the previous EF analysis had identified as a favorable scenario. We built different sets containing 10 active compounds and 1,500 decoys, all having TC < 0.3 against any actives. We used the 10 active compounds as queries and recorded the ranking of the next (second) active for each case. Finally, the 10 rankings were averaged.

### Molecular docking procedures

All docking experiments were conducted using AutoDock-GPU (Santos-Martins et al., 2021). The binding site was defined by a cubic grid of 50 Å × 50 Å × 50 Å with a spacing of 0.375 Å, centered on the known ligand-binding pocket as identified from available crystallographic structures, using the coordinates of the co-crystallized natural substrate. The receptor structure was treated as rigid throughout all simulations, while full torsional flexibility was assigned to all rotatable bonds of the ligands. Ligands were prepared using Open Babel (O’Boyle et al., 2011) and assigned Gasteiger partial charges. Each docking run consisted of 100 independent genetic algorithm (GA) searches to ensure exhaustive exploration of the binding modes and conformational space. The maximum number of energy evaluations per run was set to 2.5 × 10^6^, and other GA parameters were kept at their default values.

For each ligand, docking poses were clustered using a root mean square deviation (RMSD) cutoff of 2.0 Å. The representative binding mode was selected as the lowest energy conformation within the most populated cluster. Docking scores were computed based on the AutoDock4 scoring function (Morris et al., 2009), which combines van der Waals, electrostatic, desolvation, and torsional energy components.

Post-docking analysis included visual inspection of poses and identification of key interactions with active site residues using the software program VMD (Visual Molecular Dynamics) (Humphrey et al., 1996) and in-house Python scripts based on Biopython.

### Code availability

The set of scripts, Jupyter notebooks, and documentation used to generate, train, and evaluate the BSI models is publicly available at: https://github.com/gschottlender/bioactivity-similarity-index (MIT License).

## Results

The results are organized as follows. First, we explore the limitations when comparing compounds through chemical similarity to predict related bioactivities. Second, we design, train, and evaluate a DL-based method to predict the similarity in bioactivity (i.e., binding to the same protein, to a protein within the protein group, or to both) of chemical dissimilar compounds. Subsequently, we explore the DL method's generalization capacity by evaluating its performance for an increasingly diverse set of proteins that are not part of the training set. We subsequently extend the methodology to little-known protein groups and assess the fine-tuning of pretrained models on large and heterogeneous datasets to enhance predictive performance in protein families with limited data. Finally, we evaluated our strategy on new “unseen” ligands and in a VS-like scheme.

### Bioactivity prediction based on structural similarity: capabilities and limitations

We begin by comparing the distribution of chemical similarities, computed using the Morgan fingerprint-based TC, for both bioactive similar (S) and non-similar (N) compound pairs. Two compounds are defined as being similarly bioactive if both are defined as actives; thus, they exhibit a pChEMBL value above 6.5 for the same target (i.e., they both bind strongly to the same protein), while a pair of compounds is considered non-similar when one compound is active against a given target, and the other is not active against the same target (see Methods for details on how active non-active compounds are defined).

**FIGURE 1:**
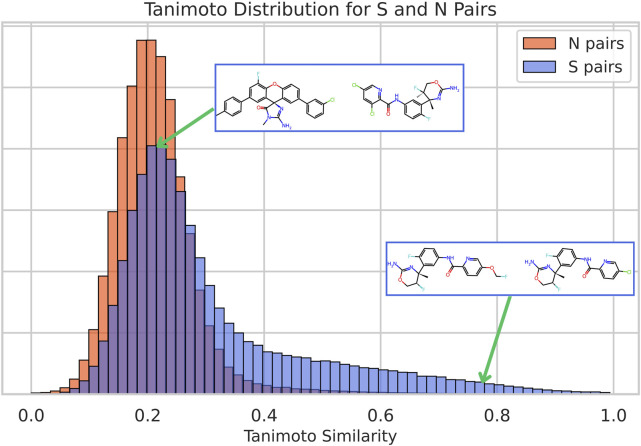
Histograms of Tanimoto similarity for pairs of compounds with similar bioactivities (S pairs) and for pairs with non-similar bioactivities (N pairs). Two different examples of S pairs for the protein P56817 are shown with their corresponding location in the distribution: a chemically similar one (compounds CHEMBL3680857 and CHEMBL3680854, with a TC of 0.78) and a chemically dissimilar one (compounds CHEMBL3695732 and CHEMBL3680890, with a TC of 0.20).

Figure 1 shows the corresponding TC histograms for the two types of compound pairs, S and N, in the whole dataset. The results show that with this “classical” methodology, using a threshold of TC of 0.4 leads, as expected, to a significant enrichment of S pairs and reflects the well-known observation that a similar chemical structure leads to similar bioactivity. However, there are many S pairs that display very low structural similarity. Indeed, 60% of S pairs have TC below 0.3, and 25% of them have TC below 0.2. Clearly, it is evident that protein binding depends on factors beyond chemical similarity. This observation underscores the fact that chemical similarity methods are robust for identifying compounds with a similar bioactivity profile in a range that nevertheless represents a minority of known compound pairs, leaving the effective comparison of structurally divergent, yet similarly bioactive, compounds as a major challenge. In the cases of our reference methods, ChemBERTa and CLAMP (Supplementary Figure S4), similarity values between compounds tend to be higher, although a substantial overlap between most S and N pairs persists.

### Evaluation of deep learning models for a bioactivity similarity index on major protein groups

To build a DL model capable of predicting bioactivity similarity between compound pairs, particularly when they are structurally different (with TC < 0.4), we used a feedforward neural network architecture, using binary cross-entropy as the loss function, and trained it to predict whether the pair of compounds belonged to the S category or not. We first trained different models for each MPG, defined as groups with more than 10 proteins harboring the same domain with reported bioactivities for more than 25 compounds. The model predictions, which correspond to the probability that the model assigns the pair as being S, and thus lie in the 0 to 1 range, will be referred to as the BSI.

Evaluations were performed following a LOPO approach, in which, for each protein in the group, the model is trained on data from all other proteins, and the excluded protein is used as the test set. Two different dataset types were evaluated, one slightly imbalanced (25:75 ratio of S to N pairs) and another heavily imbalanced (4:96 ratio of S to N). Results were compared against cosine similarity between embeddings from two state-of-the-art molecular representations, ChemBERTa and CLAMP (details in Methods).

**FIGURE 2:**
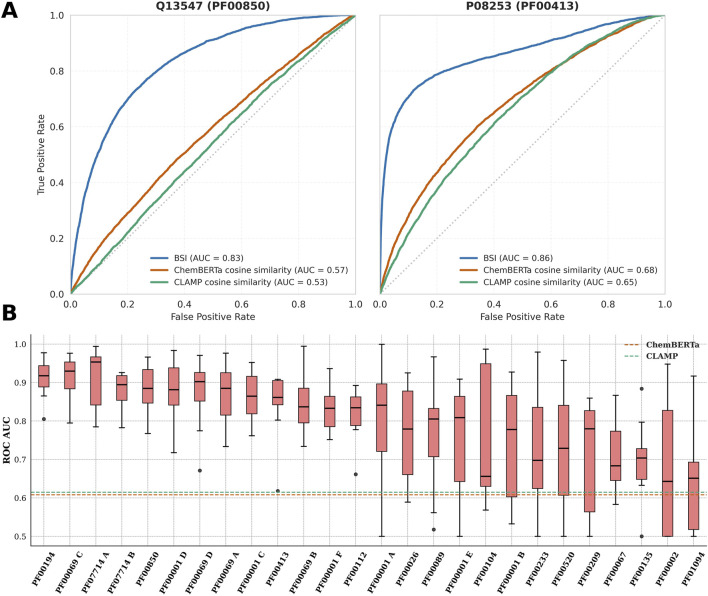
**(A)** ROC curves for BSI, ChemBERTa, and CLAMP distinguishing S from N compound pairs for proteins Q13547 and P08253 (within their respective protein groups). **(B)** Boxplots of the ROC-AUC values obtained with the BSI method for each protein group under a leave-one-protein-out evaluation. The dashed orange line indicates the mean ChemBERTa ROC-AUC across all protein groups, and the dashed green line corresponds to the mean CLAMP ROC-AUC.

Figure 2A shows the resulting ROC curves for two proteins from the MPG as representative examples (Q13547 from group PF00850 and P08253 from PF00413), evidencing superior performance of BSI over ChemBERTa and CLAMP cosine similarity, and a well-shaped ROC curve. Figure 2B shows the corresponding AUC for all MPG in descending order for the slightly imbalanced datasets. The findings indicate that BSI achieves strong predictive performance for most MPG, with AUC values above the mean value obtained using cosine similarity on ChemBERTa and CLAMP molecular embeddings, represented in the figure respectively by dashed blue and green lines. Similar results were obtained by the analysis of the precision–recall curve AUCs (See Supplementary Figure S5).

Figure 2B also shows that performance is highly variable for different MPGs. Interestingly, the following pattern is observed. For about half of the MPGs, low variability in the AUC is observed in the LOPO scheme, resulting in AUC values above 0.8, which is a very good predictive capacity. For other MPGs, the observed AUC variability is significantly higher, and the AUC values are more variable and tend to be lower. Similar results were obtained for the heavily imbalanced datasets, as shown in Supplementary Figure S6.

Overall, there appears to be some connection between data availability per protein and how well the models perform. Poorly performing protein groups—PF01094, PF00135, PF00067, PF00002, PF00520, PF00233, and PF00001 B—share the common feature of having relatively few bioactive compounds per protein (fewer than 750 bioactive compounds in total). Notable exceptions are PF00209 and PF00104, which, despite having a large amount of data per protein, exhibited considerable variability.

Conversely, PF00194 stands out as the top-performing family and also the one with the highest amount of bioactive compound data per protein. Strong results were likewise observed for PF07714 B, PF00850, PF00001 D, PF00001 C, PF00413, PF00069 B, and PF00001 F, all of which have data counts above the median. Finally, four encouraging outliers—PF07714 A, PF00069 C, PF00069 D, and PF00112—achieved good model performance despite limited data per protein. It is worth noting that PF07714 and PF00069 constitute the two main kinase clades, which may partly explain their favorable performance even under data-scarce conditions.

In the performance comparison with modern state-of-the-art models, the BSI model significantly outperformed both ChemBERTa and CLAMP (using cosine similarity) on average across all protein groups, according to paired t-tests applied to ROC-AUC and PR-AUC values. Specifically, for ROC-AUC, the differences were highly significant versus ChemBERTa (*t* = 11.99, *p* < 1 × 10^−11^) and CLAMP (*t* = 10.23, *p* < 1 × 10^−10^). Similar results were observed for PR-AUC (ChemBERTa: *t* = 10.41, *p* < 1 × 10^−10^; CLAMP: *t* = 9.86, *p* < 1 × 10^−9^).

Detailed results per test protein showed that among the 343 evaluated individual proteins (from all groups), BSI models achieved higher ROC-AUC values in 298 cases. In the remaining 55 proteins, 30 showed the best performance with CLAMP, with three of these belonging to groups PF00001 B, PF00067, and PF00233. The other 25 proteins performed better with ChemBERTa, including four cases from groups PF00233 and PF01094. All ROC-AUC and PR-AUC values obtained for each tested protein with each evaluated method are detailed in Supplementary Table S7.

### Evaluation of a single BSI model for all major protein groups (BSI-Large)

As a further assessment, we trained a single model on the full MPG data to examine whether a unified bioactivity similarity index (referred to as BSI-Large) could be established for all the evaluated clinically relevant protein families. This global index is more user-friendly, although it no longer captures group-specific activity differences.

BSI-Large was evaluated on four representative protein families chosen to cover contrasting baseline scenarios: PF00413 and PF00850, whose group-specific BSI models had performed very well, and PF00209 and PF00520, where those models had shown marked variability across proteins. Under leave-one-protein-out cross-validation, BSI-Large delivered metrics that were slightly lower (with the most substantial decrease in performance in PF00520) than the group-specific models. However, for most proteins, it still outperformed the N-versus-S discrimination achieved with ChemBERTa, CLAMP, or the Tanimoto coefficient (Supplementary Figure S8). These results show that, alongside differential indices trained for specific protein groups, a single BSI can also be developed to distinguish structurally diverse compounds.

### Assessing model generalization

Our next goal was to determine the BSI models’ ability to generalize, that is, to be able to predict similar bioactivity in proteins that are different from those used in training. The first evaluation focused on the MPG and involved using models trained for a given group to predict data for the other groups. As expected for such a challenging evaluation, overall performance was poor, with only a few predictable exceptions. Models trained on specific kinase subgroups (Pfam families PF00069 and PF07714) accurately predicted activities for other kinase subgroups. This trend did not hold for the Pfam family PF00001: models built from one subgroup of this family failed to generalize to the remaining subgroups.

**FIGURE 3:**
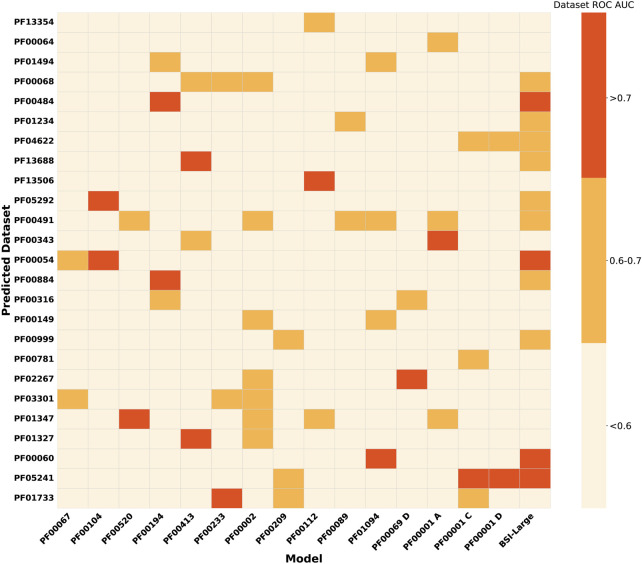
Heatmap showing the performance (mean ROC-AUC per protein) of BSI models trained for major protein groups and the general MPG model, evaluated on datasets from the underrepresented protein groups. Assessments on UPGs with at least one model prediction on the corresponding dataset with a ROC-AUC above 0.6 are shown.

As a second evaluation of the models’ generalization capacity, we analyzed their performance in protein groups that do not have enough bioactivity data to train them. We called these groups underrepresented protein groups. Figure 3 shows the performance of previously retained BSI models on 92 UPGs. The results show that for the UPGs, the performance is quite poor except for some particular cases. Interestingly, for several of the UPGs, at least one of the BSI models trained on MPG data achieved moderate or even good performance (AUC >0.6 or >0.7). For example, the model trained on bioactivity data from the PF00002 family achieved an AUC between 0.6 and 0.7 when classifying compound pairs in seven different families. Similarly, the model trained on PF00194 data reasonably predicted data from four families, with compound pairs related to the PF00484 and PF00884 families achieving an ROC-AUC greater than 0.7.

Because some of the MPG-trained models yield promising results for particular UPG and to increase the generalizability of the model, we decided to also evaluate the BSI-Large, which was built by merging all MPG data in a single training set, as previously explained. The model was evaluated on the UPG, and the results are presented in the last column of Figure 3. The BSI-Large model, as expected, shows a better overall performance and is in many cases at least as good, or even better, than the best MPG-trained model. However, for some particular UPG, the model still shows poor performance.

These results suggest that models trained with larger and more diverse protein sets can partially generalize and understand the underlying features that make two compounds display similar bioactivities in a wide range of protein targets, without compromising the higher predicting capacity achieved with a more focused training.

### Evaluations in protein groups with minimal data availability and the applicability of transfer learning to enhance predictive performance

Another potential approach to improve BSI model transferability, that is, its capacity for accurately predicting similar bioactivity in unseen protein groups, is based on the transfer learning strategy (Sevakula et al., 2019; Cai et al., 2020). In this case, the BSI-Large, the more general model, is fine-tuned on protein groups with very limited data. Fine-tuning is carried out by performing a second training of the BSI-Large model using new data from each UPG for five additional epochs (detailed in Methods).

**FIGURE 4:**
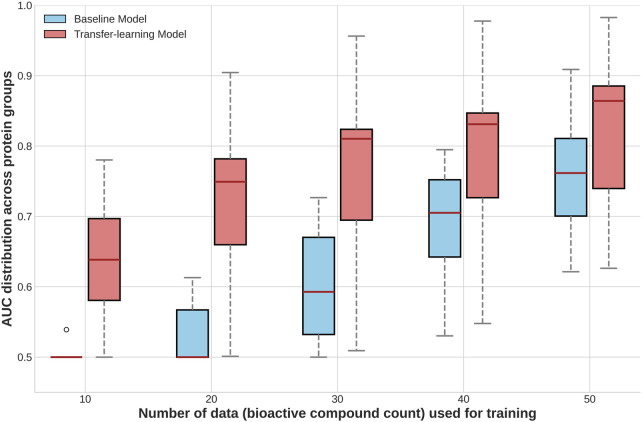
Boxplots comparing the distribution of performance (ROC-AUC per group) of models trained with and without transfer learning, across different training-test splits for datasets of less represented protein groups.

We evaluated the performance of the fine-tuned BSI-Large model on several UPGs and compared it to the baseline BSI-Large model. The results, presented in Figure 4, demonstrate that fine-tuning (or transfer learning) substantially enhances the model’s predictive capacity. For example, with less than 20 active compounds, the baseline model performs poorly, but the fine-tuned model already achieves over 0.7 ROC-AUC. As expected, as more “unseen” data are used to fine-tune the model, the performance increases but tends to plateau. It is also interesting to note that additional data increases the performance of the baseline model. However, the fine-tuned general model still outperforms it. Overall, these results highlight first, the possibility of training models based on a small dataset that generate an effective BSI capable of accurate predictions over a much larger universe. Second, it underscores the contribution of transfer learning to enhance predictive performance in scenarios with very limited training data, which is especially promising in cases of scarce information.

### Validation on compounds with corresponding bioactivities recently determined by experimental methods

For subsequent validation using experimentally verified results, we selected pairs of compounds from ChEMBL version 35 that were not present in the training dataset (version 33). We analyzed the performance of different models on 109 proteins with new data, 88 of them belonging to 19 different MPG and 21 proteins from 16 distinct UPGs, selecting only those compound pairs with a TC < 0.3. We complemented the S pairs with N pairs using decoys, using a heavily imbalanced approach (2.25:97.75 ratio of S/N) to better reflect a realistic scenario, where only a small fraction of active compounds is typically found within a much larger database. The mean enrichment factor at the top 2% (EF_2%_) was evaluated and compared with ChemBERTa and CLAMP for each protein group, averaging across all proteins within the group.

For proteins belonging to the MPG, we evaluated both the group-specific BSI models and the BSI-Large model. The group-specific models achieved a mean EF_2%_ greater than 5 in 10 protein groups, while for the remaining groups, the mean EF_2%_ exceeded 2. In the case of the BSI-Large model, a mean EF_2%_ above 5 was observed in only six groups; in another nine groups, the mean EF_2%_ ranged between 2 and 5, and in the remaining three groups, it was below 2. As expected, the TC showed no enrichment in S pairs (mean EF_2%_ ≤ 1.0) for most groups (except for PF00067, which achieved an EF_2%_ of 1.96), reflecting its limited ability to recover true S pairs among dissimilar compound pairs. ChemBERTa similarity showed enrichment (EF_2%_ > 1.0) in 17 groups, ranging from 1.04 (PF00067) to 1.98 (PF00194), thus outperforming TC overall. CLAMP similarity yielded enrichment in 14 groups, with EF_2%_ values above 2 for six of them: PF00001 C (3.39), PF00001 D (2.61), PF00001 E (2.63), PF00069 C (2.73), PF00089 (3.12), and PF00209 (2.57), indicating a significant improvement, although still considerably lower than the BSI models.

**FIGURE 5:**
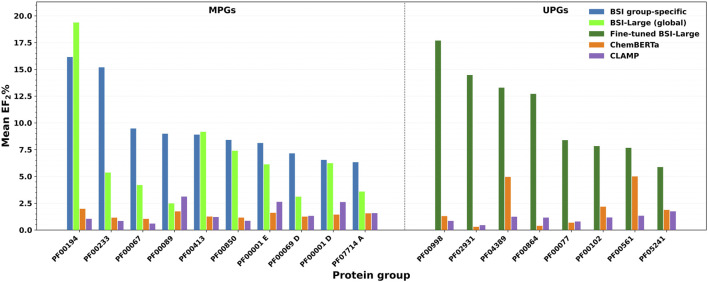
Enrichment factor at the top 2% (EF_2%_) for S pairs. On the left: data from the MPGs evaluated with the group-specific BSI model (blue), the global BSI-Large model (green), ChemBERTa (orange), and CLAMP (purple). Right panel: data from the UPGs assessed with a BSI-Large model fine-tuned on UPG data up to ChEMBL 33 (magenta), ChemBERTa (orange), and CLAMP (purple).

Overall, the best performance was achieved in fourteen protein groups with the group-specific BSI models, while the remaining five groups showed the highest enrichment with the general BSI-Large model. None of the evaluated groups exhibited better performance for ChemBERTa or CLAMP, although CLAMP outperformed the BSI-Large (but not the group-specific BSI) in two cases (PF00069 C and PF00089). Statistical analysis further supported that both the group-specific BSI and general BSI-Large models achieved significantly higher enrichment than ChemBERTa and CLAMP. For the group-specific BSI models, the differences in EF_2%_ were highly significant versus ChemBERTa (*t* = 6.08, *p* < 1 × 10^−5^) and CLAMP (*t* = 5.36, p < 5 × 10^−5^). For the BSI-Large model, the improvement remained significant (ChemBERTa: *t* = 3.58, *p* = 0.002; CLAMP: *t* = 3.07, *p* = 0.007), supporting the overall robustness of the enrichment performance across MPG. Representative examples of the most promising BSI predictions in comparison with ChemBERTa and CLAMP for the MPG are shown in Figure 5 (left panel).

For the proteins in the UPG, we evaluated a BSI-Large model that was fine-tuned using all available data across the corresponding protein groups. The fine-tuned BSI-Large model achieved a mean EF_2%_ larger than 5 in eight groups, with two of these groups reaching values above 10. In six additional groups, the mean EF_2%_ ranged between 2 and 5, and in the remaining two groups (PF00017 and PF07690), the model showed poor performance, with mean EF_2%_ values close to 0. Similar to the evaluation performed for the MPG, the TC lacked predictive power, exhibiting no enrichment. ChemBERTa similarity showed enrichment in 11 groups, with EF_2%_ values above 2 in four of them: 5.00 for PF00561, 4.96 for PF04389, 4.25 for PF00248 (an interesting case where ChemBERTa outperformed the fine-tuned BSI-Large, which showed an EF_2%_ of 2.64), and 2.18 for PF00102. CLAMP similarity also yielded enrichment in 11 groups, but only two displayed EF_2%_ values greater than 2—PF00248 (3.07) and PF04622 (2.10).

Taken together, in the UPG, the fine-tuned BSI-Large retrieved the best performance in 13 groups. ChemBERTa performed better on two groups (PF00017, where the fine-tuned BSI-Large showed no enrichment, and PF00248), while none of the methods achieved enrichment in the remaining group (PF07690). Statistical analysis supported that higher EF_2%_ values were achieved using fine-tuned BSI-Large than both ChemBERTa (t = 6.08, *p* < 1 × 10^−5^) and CLAMP (t = 5.36, *p* < 5 × 10^−5^), confirming that the improvements remain statistically robust even in data-scarce conditions. Representative results for groups with mean EF_2%_ values above 5 are shown in Figure 5 (right panel), compared with the results from the other two methods.

As an additional case study in a VS-like setting, we selected the α2B adrenergic receptor (ADRA2B; UniProt P18089) that belongs to Pfam family PF00001, subgroup D. In our global evaluation, this subgroup consistently showed good EF_2%_ values. For the experiment, we built a library of 1,500 chemically diverse compounds, of which only 10 were confirmed ADRA2B actives (≈0.7% prevalence), and all pairs have TC < 0.3. Using each of the 10 active ligands as the query ligand, we ranked the whole library by similarity, recorded the rank of the next active, and averaged over all ten queries. TC showed an average rank of 45.2 (range: 1–205). Among the evaluated models, ChemBERTa retrieved an average rank of 54.9 (range: 1–288), while CLAMP performed better, with a mean rank of 28.6 (range: 1–107). In contrast, the BSI group-specific model further reduced the rank to 3.9 (range: 1–17), and the BSI-Large model to 10.5 (range: 1–88).

These results can be interpreted as follows. Given a known active used as the query, a TC-based search would require testing ∼45 compounds to find one new binder with a different chemotype, ChemBERTa would require testing ∼55 compounds, and CLAMP would require testing ∼29 compounds. In contrast, BSI requires testing fewer than 15. It is interesting to note that the BSI models also retrieved more remote chemotypes: the first active recovered by the group-specific model had a mean TC of 0.21 to the query, and BSI-Large had a TC of 0.18, whereas the TC similarity search itself yielded a less-dissimilar first hit with a mean TC ≈ 0.26. ChemBERTa and CLAMP likewise yielded first active hits with a low TC (0.22 and 0.20, respectively), showing that these embedding-based methods can provide low-similarity actives that remain undetected in a TC-only search.

### Illustrative examples of the BSI model’s predictive capacity

**FIGURE 6:**
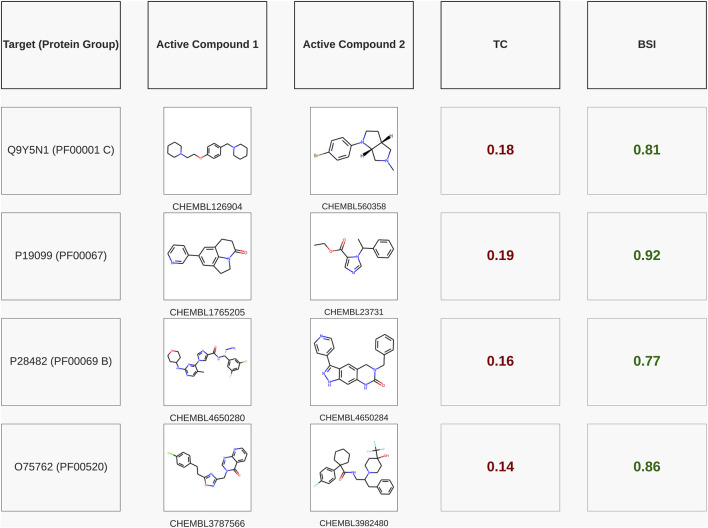
Pairs of active compounds for the same test targets (with their respective Pfam families) that exhibit a very low TC, showing that the BSI predicted with the model trained for the corresponding protein group achieved a high similarity value. The targets are named with their UniProt IDs.

To further illustrate the predictive capacity of BSI, we selected four representative examples of compound pairs that are known to bind the same protein target according to ChEMBL yet exhibit very low structural similarity (TC < 0.2). Figure 6 shows that in all these cases, BSI assigns high similarity values (BSI >0.75), successfully capturing their shared bioactivity despite the lack of obvious structural resemblance. The first target corresponds to the human H_3_ receptor (UniProt Q9Y5N1). For the human H_3_ receptor (UniProt Q9Y5N1), the pair CHEMBL126904 (diphenylalkylamine, diaryl-ether–piperidine) and CHEMBL560358 (tropane derivative) shows a very low structural similarity (TC = 0.18), yet BSI recognizes their shared activity, assigning a high score of 0.81 (pChEMBL 8.05 and 7.0). For CYP11B2 (UniProt P19099), CHEMBL1765205 (quinolinone derivative) and CHEMBL23731 (imidazole ester, etomidate-like) share a TC of 0.19 but achieve a BSI of 0.92 (pChEMBL 8.96 and 10.0). For MAPK1 (UniProt P28482), the pair CHEMBL4650280 (indazole carboxamide) and CHEMBL4650284 (quinazolinone derivative) displays a TC of 0.16 while reaching a BSI of 0.77 (pChEMBL 8.7 and 8.0). Finally, for TRPA1 (UniProt O75762), CHEMBL3787566 (diaryl-azole carboxamide) and CHEMBL3982480 (diarylalkylamine, piperidine type) exhibit a TC of only 0.14, yet BSI assigns a strong similarity score of 0.86 (pChEMBL 6.81 and 8.4).

**FIGURE 7:**
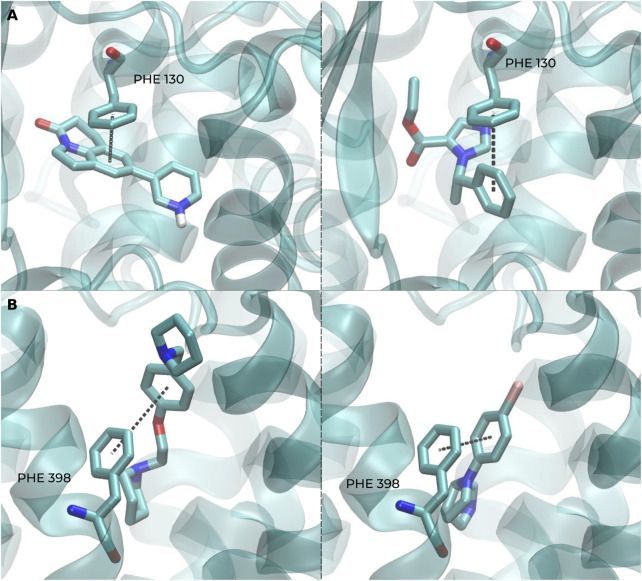
Representative docking poses highlighting key ligand–protein interactions captured by BSI-predicted pairs. **(A)** Docking shows a π–π interaction (PHE 130) and a hydrogen bond (LEU 451) in CYP11B2 for both CHEMBL1765205 (left) and CHEMBL23731 (right). **(B)** Similarly, both CHEMBL126904 (left) and CHEMBL560358 (right) form aromatic interactions with residues PHE 398 and TRP 110 of the human H_3_ receptor.

For a better biological interpretation of the results obtained using the BSI, we analyzed the molecular interactions from two representative S pairs using molecular docking. In the first case, involving CHEMBL1765205 and CHEMBL23731, both ligands establish the key interaction with CYP11B2 through a pi-stacking (aromatic) interaction with PHE 130 and a hydrogen bond with LEU 451 in Figure 7A. Similarly, in the second example, the S pair CHEMBL126904/CHEMBL560358 forms aromatic interactions with residues PHE 398 and TRP 110 of the human H_3_ receptor (Figure 7B). These findings suggest that, in these examples, compounds with a low Tanimoto coefficient but a high BSI can share a similar action mechanism against the same protein, forming key interactions with certain identical amino acid residues.

## Discussion

Predicting similar chemical or biological activities between chemical compounds represents an important challenge. It is a highly valuable tool in a wide range of applications, including drug discovery. Structural similarity, commonly computed using the Tanimoto coefficient between molecular fingerprints, as well as other structure-based metrics, is a useful tool providing confident results, as shown in the first part of our work. However, its applicability is limited to a certain similarity threshold, and it also fails to capture the inherent complexities of molecular interactions. This leaves a practical blind spot: many functionally related ligands fall below similarity cutoffs of commonly used methods (e.g., TC < 0.30), narrowing ligand-based discovery and limiting the exploration of structurally remote chemotypes. Accordingly, new approaches are needed to address the problem of discovering novel bioactive compounds that cannot be identified through the conventional structural compound similarity approach. Using machine learning approaches for identifying compounds with similar bioactivities for the inhibition of different specific targets has become a common practice in recent years (Park et al., 2022; Shin et al., 2022; Hadipour et al., 2025). Methods designed to find structurally dissimilar compounds based on similar bioactivity profiles have been developed (Petrone et al., 2012; Yu et al., 2015), as well as those based on target similarity (Periwal et al., 2022). However, a reference benchmark is still missing. Recently, advanced deep learning architectures have been introduced to learn molecular similarity directly from pairs of compounds, providing an alternative to traditional structure-based metrics (Fernández-Llaneza et al., 2021). While such models successfully captured bioactivity relationships beyond Tanimoto-based similarity, they were trained and evaluated on single-target datasets. Here, we extend this concept to a protein-group framework that learns generalizable bioactivity similarity patterns across one or multiple protein families.

In this study, we show that when using deep learning-based techniques with molecular fingerprint tokenization, it is possible to classify pairs of compounds that are highly different in structure (TC < 0.3) but exhibit similar bioactivities. In other words, they bind the same (or similar) proteins. Training using individual protein groups yields models that can reach very high accuracies (AUC >0.8–0.9), but the performance is quite system dependent, and low transferability is obtained. Training a general model with combined information significantly increases transferability and diminishes the predictive capacity variance between different protein groups. Moreover, fine-tuning using group-specific data boosts accuracy to very high levels. Transfer learning allows model fine-tuning in protein groups with a limited set of compounds, with 20 actives already providing moderate to high accuracies. Overall, across our evaluation datasets, BSI consistently improved early retrieval (top 2% enrichment factor, EF_2%_), recovered low-TC functional analogs, and enabled transfer learning for underrepresented families, compared with structure-based similarity and modern embedding baselines (ChemBERTa, CLAMP; cosine similarity), resulting in a useful complement to conventional metrics.

Although it remains challenging to develop a trained similarity index that functions analogously to the TC across the entire universe of chemical compounds, the BSI-Large model constitutes a first step by integrating information from diverse protein families. There is considerable room for improvement through more advanced data engineering and the incorporation of sophisticated architectures and molecular descriptors. However, this approach proved particularly effective when applied using group-specific models—which outperformed the global model across our evaluations—in scenarios where the biological system is known. In practice, this is a common situation: researchers usually seek similar bioactive compounds for a defined target system, making the application of group-specific models (MPG or generated for UPG by fine-tuning BSI-Large) especially relevant (Wang et al., 2022). For example, this approach can identify additional bioactive compounds for an understudied protein belonging to an MPG or UPG, even when only one or two known bioactive compounds are known. The Tanimoto coefficient (or ChemBERTa, CLAMP, and other related methods) can be used to retrieve structurally similar actives, while BSI can serve as a complementary tool to detect potentially active but structurally dissimilar compounds. Furthermore, this methodology can be extended to the study of differential activity, that is, to determine whether the bioactivity of compound pairs varies across different protein groups. As previously mentioned, these methods are intended to complement conventional metrics, particularly below their confidence threshold.

Although our primary aim was to search for similar bioactive compounds, our index could also be used as an alternative (or complement) to the TC in other applications that require comparing chemical compounds. Comparison of chemical compounds is usually used, for example, to build diverse chemical datasets for testing in experimental and/or virtual high-throughput screening campaigns. In this scenario, instead of using the TC, compounds could be selected to reflect a more diverse set in terms of our herein developed BSI, which could aim to have, for a given set size, a more diverse dataset in terms of their potential bioactivity. Another potential use of the BSI is to identify compounds with desired ADMET (absorption, distribution, metabolism, excretion, and toxicity) properties that are predicted to have similar bioactivities to a known active compound with a problematic ADMET profile. In this case, our approach offers additional potential to relying on the TC because high TC often tracks ADMET similarity, whereas our method can recover bioactivity relationships that TC misses.

### Future work

Possible alternative bioactivity-related applications correspond to the discovery of enzyme substrates in biotechnology, where interchangeable molecules for biocatalysis or metabolic engineering often escape 2D fingerprint searches (Kroll et al., 2023; Schottlender et al., 2024). As alternative approaches beyond bioactivity, BSI-like trained indices can assist in identifying replacement chemicals, such as solvents, plastics, or industrial additives, by detecting compounds that—despite low structural similarity—share key properties (Thouand et al., 2011; Damayanti et al., 2015; Bystrzanowska and Tobiszewski, 2020; Driver and Hunter, 2020). In this way, we propose our approach as a starting point for developing trained compound similarity indices tailored to specific objectives.

As future perspectives, the developed methods present a wide margin for improvement. First, it is possible to incorporate more advanced molecular representations, such as learned embeddings (for example, generated using models based on Transformers), graph architectures, or even fingerprints of greater length or different types (Sabando et al., 2022; Yi et al., 2022; Luong and Singh, 2024). Although this work opted for a simple and efficient 256-bit representation, adopting more sophisticated alternatives (such as ChemBERTa or CLAMP, higher-dimensional embeddings that showed reasonable enrichment of active compound pairs when using cosine similarity as a compound comparison metric) could result in a performance boost, especially when combined with deeper and more complex neural network architectures, provided that sufficient computational resources are available. For example, attention-based multimodal fusion has shown improvements in Natural Products (NP) anticancer prediction and could be adapted to our bioactivity similarity setting (Norouzi et al., 2025). Additionally, capsule-inspired part–whole encoders provide a transferable architectural prior we could test to strengthen retrieval under low structural similarity (Abbasi and Razzaghi, 2020).

Second, dataset engineering and curation constitute a key aspect, especially for models such as BSI-Large, which are trained on data from numerous protein groups. As observed, this diversity can introduce noise and limit predictive capacity compared to models specific to each group. Further research focused on data selection, processing, and balancing could enable the development of more robust global models, applicable to broader contexts such as phenotypic screening (Zheng et al., 2013; Xia, 2017). Finally, one of the most relevant challenges in this type of approach is the scarcity of reliable negative data as these are typically not reported or published in the literature. Improving the availability of information on inactive compounds and advancing data augmentation techniques specifically for negative examples would help increase the robustness and applicability of the models developed (An et al., 2025).

## Conclusion

We presented BSI, a learned, pairwise, bioactivity-centered similarity model defined across protein families and explicitly trained on structurally dissimilar pairs (e.g., TC < 0.30–0.40). A global, multi-family variant (BSI-Large) remains competitive across families and supports transfer learning for underrepresented protein groups. Relative to single-target pairwise approaches, our design is novel in both its low-TC focus and its evaluation protocol: a LOPO scheme, demonstrating applicability to proteins that were not encountered during training. BSI complements structure-based metrics and embedding baselines (ChemBERTa, CLAMP; cosine similarity) by recovering structurally dissimilar functional analogs and improving early retrieval (EF_2%_) on retrospective benchmarks. While current coverage is limited to a clinically relevant subset of targets, the approach offers a practical path toward broader adoption through richer representations, new training data, and fine-tuning or domain adaptation.

## Data Availability

Publicly available datasets were analyzed in this study. These data can be found at: ChEMBL database, version 33: https://ftp.ebi.ac.uk/pub/databases/chembl/ChEMBLdb/releases/chembl_33 ChEMBL; ChEMBL database, version 35: https://ftp.ebi.ac.uk/pub/databases/chembl/ChEMBLdb/latest/.
