# Supplementary figures and images for: Beyond Tanimoto: a learned bioactivity similarity index enhances ligand discovery
Source: Front Bioinform. 2025 Nov 28;5:1695353. doi: 10.3389/fbinf.2025.1695353 (PMC12698616; doi:10.3389/fbinf.2025.1695353)

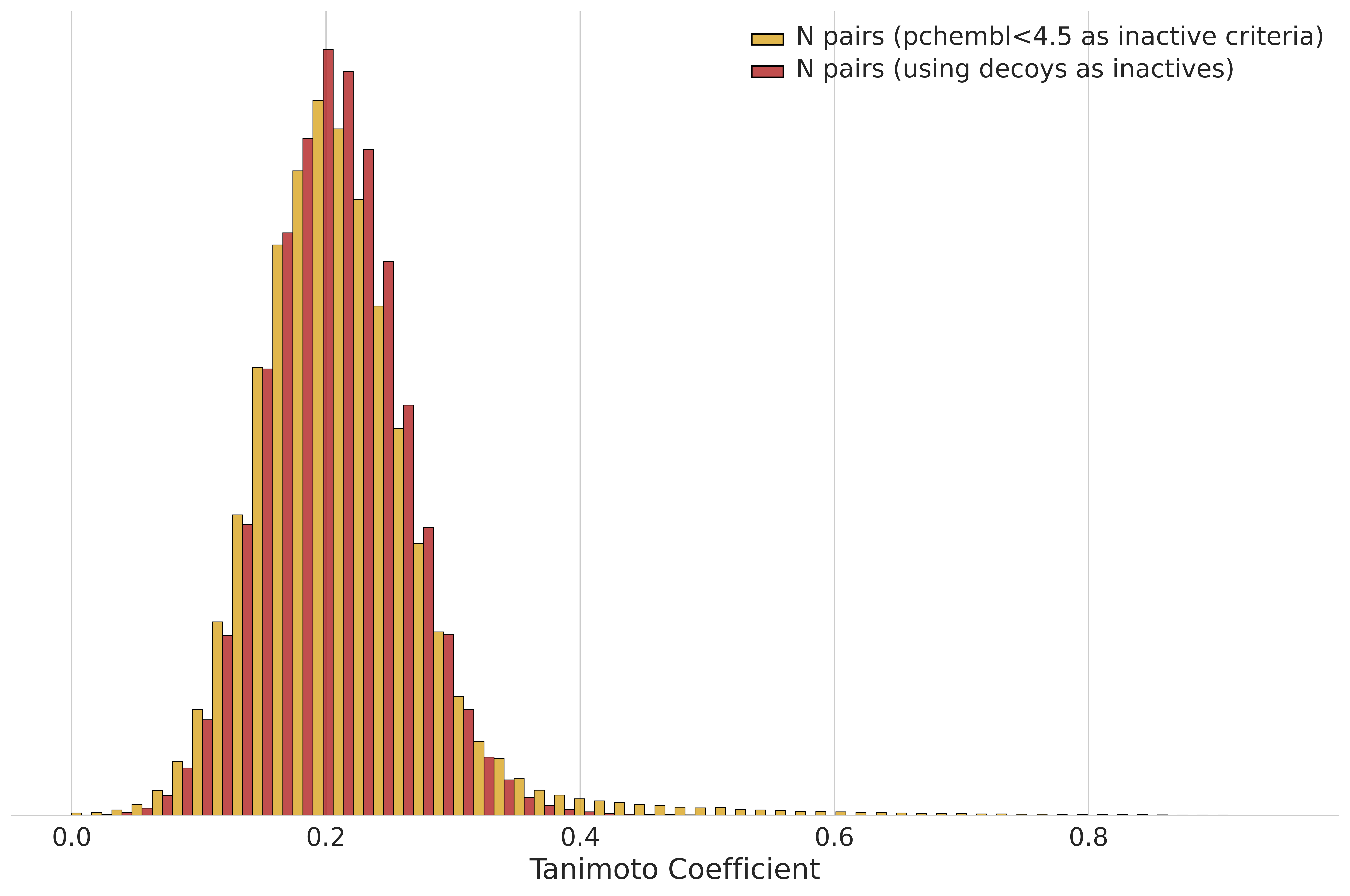

Supplement: Supplementary file 1 [file DataSheet1.zip › S1.png]

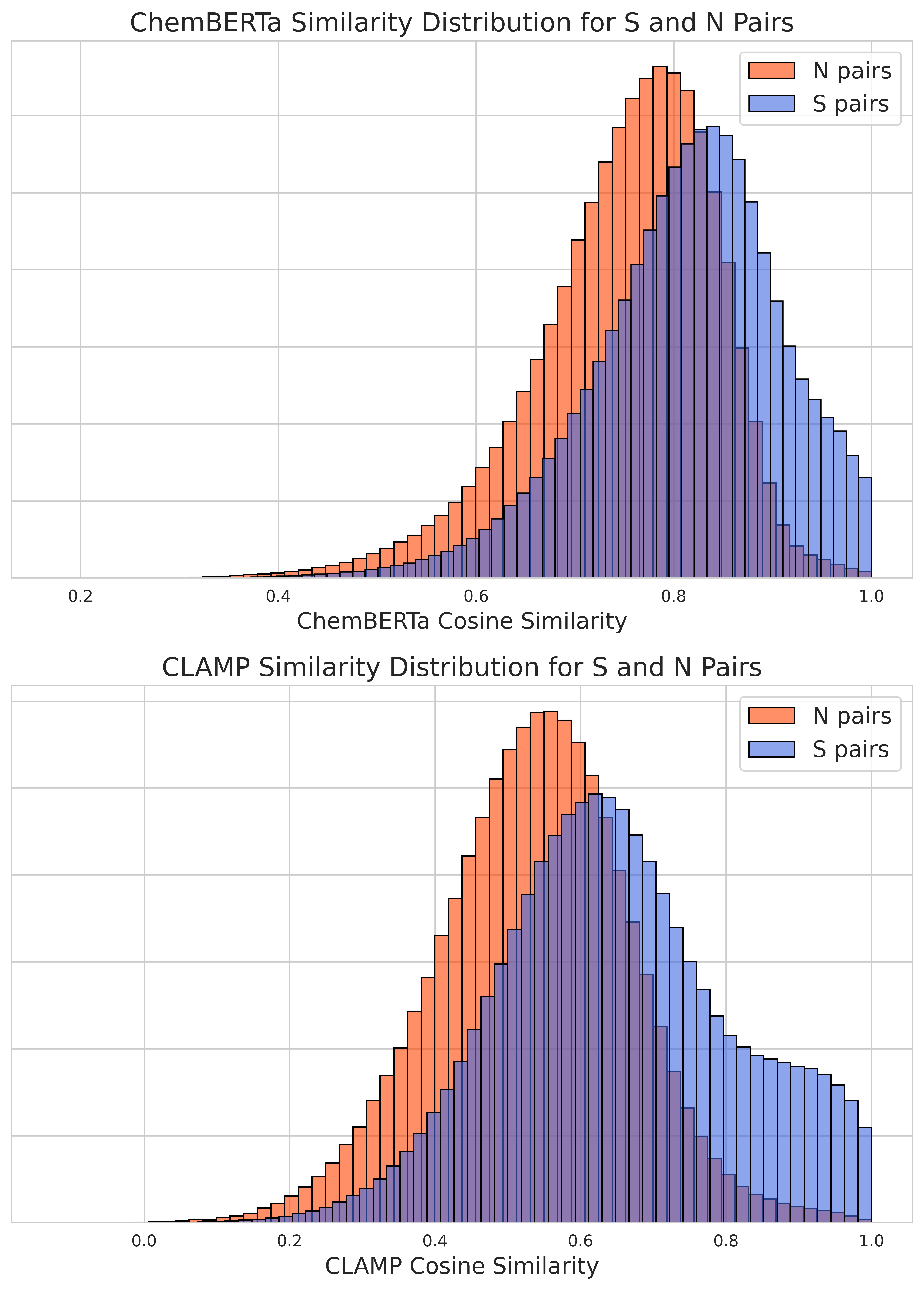

Supplement: Supplementary file 1 [file DataSheet1.zip › S4.png]

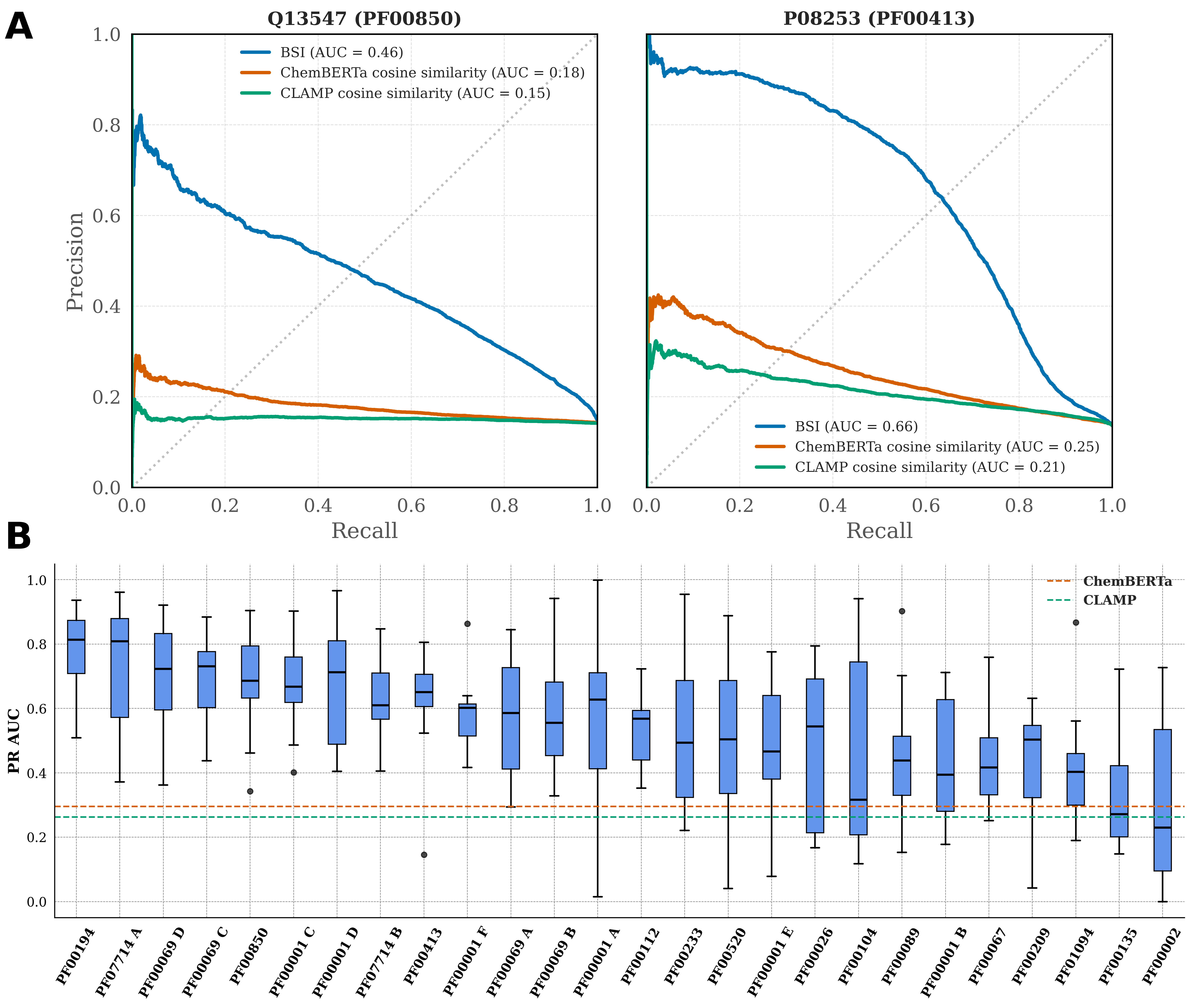

Supplement: Supplementary file 1 [file DataSheet1.zip › S5.png]

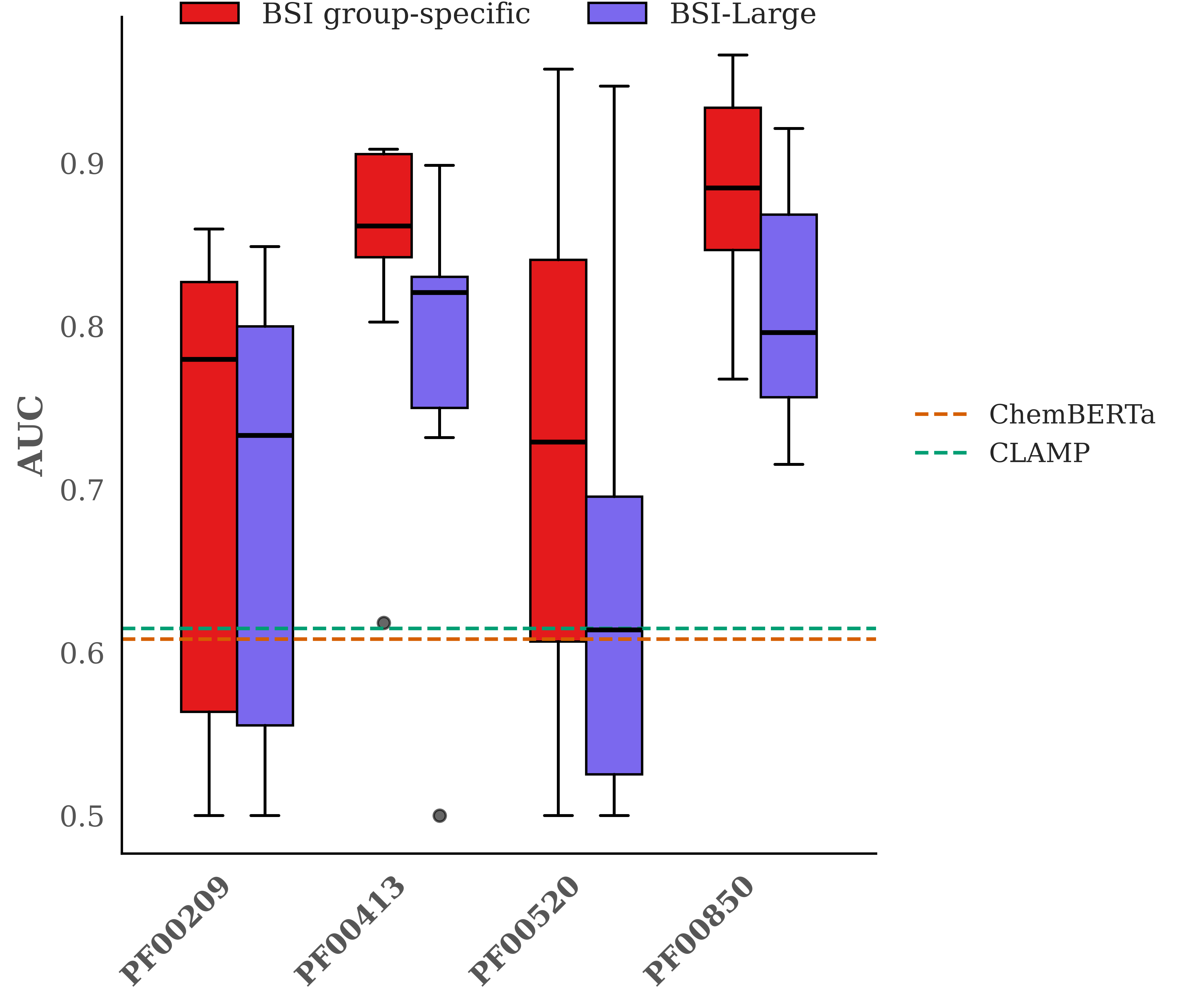

Supplement: Supplementary file 1 [file DataSheet1.zip › S8.png]
